# Urolithin A Attenuates Periodontitis in Mice via Dual Anti-Inflammatory and Osteoclastogenesis Inhibition: A Natural Metabolite-Based Therapeutic Strategy

**DOI:** 10.3390/molecules30132881

**Published:** 2025-07-07

**Authors:** Yishu Xia, Danni Wu, Linyi Zhou, Xinyu Wu, Jianzhi Chen

**Affiliations:** 1 School of Stomatology, Zhejiang Chinese Medical University, Hangzhou 310053, China; 202211125011023@zcmu.edu.cn (Y.X.); 202211125011032@zcmu.edu.cn (L.Z.); 2 Hangzhou Medical College, Hangzhou 310053, China; wudanni@hmc.edu.cn; 3 School of Basic Medical Science, Zhejiang Chinese Medical University, Hangzhou 310053, China; 15868905221@163.com

**Keywords:** Urolithin A, periodontitis, bone resorption, inflammation

## Abstract

Periodontitis is an inflammatory disease that affects the periodontal supporting tissues. Its cardinal clinical manifestations encompass gingival inflammation, periodontal pocket formation, and alveolar bone resorption. Urolithin A (UA), a gut microbiota-derived metabolite of ellagitannins, is known for its anti-inflammatory and osseous-protective properties. Nonetheless, the impact of UA on periodontitis remains unknown. To investigate the preventive effect of UA, we employed a lipopolysaccharide (LPS)-induced inflammation model in RAW 264.7 mouse macrophages, a receptor activator of nuclear factor-κB ligand (RANKL)-induced osteoclast differentiation model, and a ligature-induced periodontitis model in mice. The expression of inflammatory factors (tumor necrosis factor-α, TNF-α; interleukin-6, IL-6) was analyzed to assess anti-inflammatory efficacy. Bone loss in mice with periodontitis was assessed through histological and imaging techniques, including haematoxylin and eosin staining to evaluate alveolar bone morphology, Masson’s trichrome staining to visualize collagen fiber distribution, and micro-computed tomography scanning to quantify bone structural parameters. Additionally, we investigated the underlying mechanisms by examining osteoclast activity through tartrate-resistant acid phosphatase staining and the expression levels of proteins RANKL and osteoprotegerin (OPG). We found that UA reduced IL-6 and TNF-α levels in vitro and in vivo, inhibited osteoclast differentiation, and decreased the RANKL/OPG ratio in periodontitis mice.

## 1. Introduction

Periodontitis is a chronic, multifactorial inflammatory disease that is attributed mainly to the accumulation of dental plaque biofilm. Owing to the presence and persistence of dental plaque biofilm, an immune-inflammatory response may be triggered in the host to destroy periodontal supporting tissues (e.g., periodontal ligament and alveolar bone) progressively. A failure of timely control of this pathological process may result in tooth mobility and even loss [1]. According to existing longitudinal studies over the past three decades, there has been a continuous increase in the prevalence of severe periodontitis. As of 2019, 1.1 billion people worldwide were estimated to be affected by severe periodontitis [2].

There are various therapies available for periodontal treatment, including behavioral correction methods such as personalized oral hygiene instructions, smoking cessation support, and dietary changes. Other clinical interventions include scaling and root planing to remove dental plaque and calculus, laser-assisted therapies [3], periodontal tissue regeneration [4], and local or systemic antibiotics [5]. Currently, systemic antibiotics remain a wide option due to their broader antimicrobial coverage and established clinical outcomes despite targeted delivery and reduced systemic exposure provided by the use of local antibiotics [6]. Common systemic antibiotics include doxycycline [7], azithromycin [8], the Van Winkelhoff cocktail (amoxicillin + metronidazole) [9], etc. Despite all this, systemic antibiotics, in case of overuse and inappropriate application, can induce antibiotic resistance and the development of multidrug-resistant bacterial strains [10]. Prior studies have reported a temporary increase in the proportion of antibiotic-resistant oral bacterial species, even after a single round of systemic antibiotic therapy [11,12]. Moreover, previous epidemiological studies have documented a significant exposure-response relationship regarding antibiotic resistance in periodontal pathogens, with relatively higher resistance rates in patients who used systemic antibiotics frequently [13]. Therefore, this highlights the significance of identifying a safe, effective, anti-inflammatory, and osteogenic drug for both oral clinicians and researchers.

**Figure 1:**
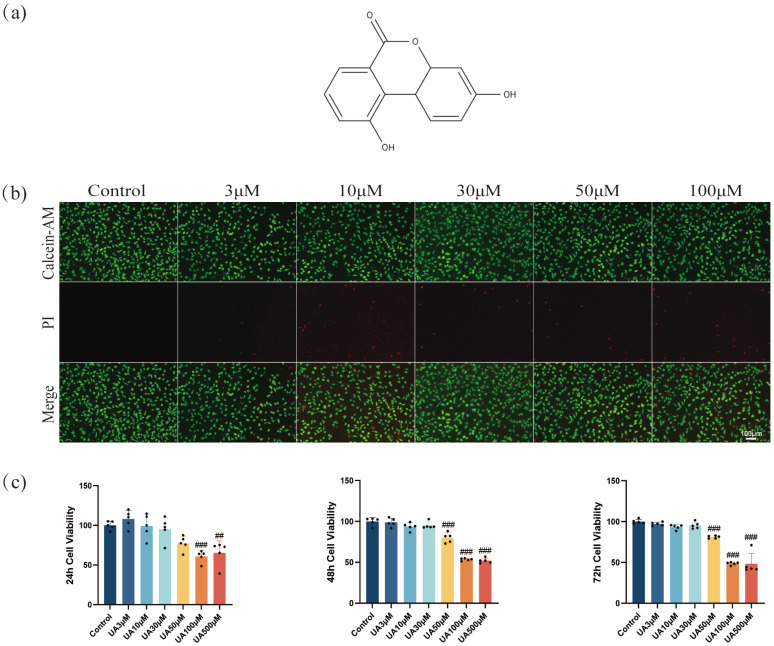
Effects of UA on cell viability. (**a**) Chemical structure of UA (C_13_H_8_O_4_, molecular weight: 228.20 g/mol). (**b**) Cell death was assessed by Calcein-AM/PI staining. (**c**) The cytotoxicity of UA on L929 cells was assessed using the CCK-8 assay at different concentrations over 24, 48, and 72 h. Data are presented as the mean ± SD. ## *p* < 0.01, ### *p* < 0.001 compared with the control group. UA, Urolithin A; PI, propidium iodide.

Urolithin A (UA) (3,8-dihydroxybenzo[c]chromen-6-one) (Figure 1a), belonging to the family of urolithins, can be structurally classified as a benzo[c]coumarin-based compound. The biosynthesis of this compound primarily relies on the metabolic conversion by the gut microbiota. Specifically, after oral intake and ingestion of dietary polyphenols (e.g., ellagic tannins and ellagic acid) sourced from pomegranate, strawberries, raspberries, and walnuts, these components are further gradually transformed into urolithin compounds in the colon by specific bacterial species (e.g., *Gordonibacter pamelaeae* DSM 19378T and *Gordonibacter urolithinfaciens* [14]). Emerging data has elucidated many pharmacological effects of UA as research deepens and relevant mechanisms are explored. For example, UA showed good safety through a series of standardized toxicological assessments, including a 90-day subchronic exposure study in rodent models [15]. Moreover, clinical trials in humans have also demonstrated a favorable safety profile of UA, with no report of serious adverse events or drug-related nonserious adverse events [16]. Meanwhile, UA has been proven to have anti-inflammatory [17,18], anticancer [19,20], and anti-aging [21,22,23,24] effects. Recent research on oral health has revealed the effective role of UA in suppressing the release of pro-inflammatory cytokines [e.g., interleukin (IL)-1β (IL-1β), IL-6, and tumor necrosis factor-α (TNF-α)] in macrophages infected with Porphyromonas gingivalis, by activating the PINK1-mediated mitophagy pathway [25]. In addition, ellagic acid and ellagic tannins have been confirmed to have inhibitory effects on osteoclast differentiation [26]. On this basis, it may be theoretically feasible to develop natural product-based bone metabolism regulators, which have shown potential applications, particularly in the treatment of alveolar bone resorption associated with periodontitis. As a gut metabolite of the natural phytochemicals ellagitannins (ETs), UA has attracted considerable attention in recent decades. At the current stage, little is known about the specific mechanisms of UA in the treatment of periodontitis, despite its great potential in treating cancer [19,20], cardiovascular diseases [27,28], neurodegenerative disorders [29,30], and metabolic dysfunctions [31,32]. In particular, further systematic investigation is required to decipher the dual effects of UA on regulating inflammation and balancing bone metabolism in experimental periodontitis models.

Accordingly, the present study was designed to investigate the therapeutic effects and underlying mechanisms of UA in a mouse model of experimental periodontitis. This study systematically evaluated the effects of UA on inflammatory responses, osteoclast differentiation, and alveolar bone microarchitecture both in vitro and in vivo. This study is anticipated to provide new theoretical insights and potential strategies for targeted therapy of periodontitis.

## 2. Results

### 2.1. Effects of UA on L929 Viability

The CCK-8 assay results showed that UA exhibited a concentration- and time-dependent cytotoxic effect on L929 cells (Figure 1c). UA (3–30 µM) exhibited no significant cytotoxicity toward L929 cells after 24, 48, or 72 h of treatment. However, cell viability decreased after treatment using ≥50 µM UA. Notably, at the same concentration, as the treatment time extended from 24 h to 72 h, the cell viability showed a gradual decline, suggesting a time-dependent impact of UA at high concentrations on cell viability. Therefore, UA showed low cytotoxicity at ≤30 µM but induced significant toxicity to L929 cells at ≥50 µM. Furthermore, live/dead cell staining confirmed the concentration-dependent cytotoxic effects of UA. Specifically, as the concentration of UA increased from 3 µM to 100 µM, the proportion of PI-positive cells (dead cells) increased significantly (Figure 1b). This result was highly consistent with the CCK-8 assay findings, confirming that UA was toxic to L929 cells at ≥50 µM. Based on these findings, to ensure experimental reliability and cell viability, UA at 3, 10, and 30 µM were determined to be used for subsequent experiments, suggesting no significant cytotoxicity and exhibiting potential biological activity.

### 2.2. Effect of UA on Inflammation in a Periodontitis Model

**Figure 2:**
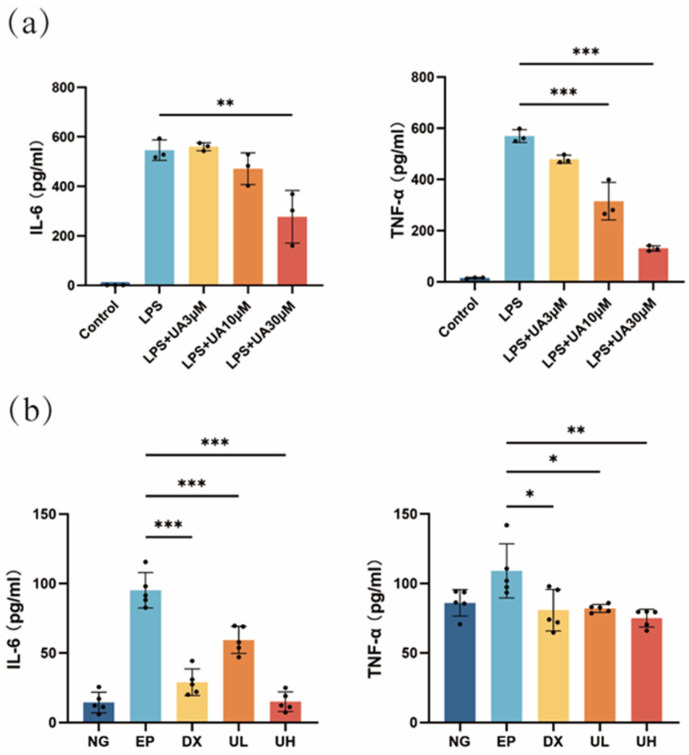
Effects of UA on inflammatory cytokine levels. (**a**) RAW264.7 cells produce IL-6 and TNF-α in response to LPS stimulation, and the results show the effect of UA on the production of these inflammatory cytokines. (**b**) Impact of UA on serum levels of TNF-α and IL-6 in mice with experimental periodontitis. Data are presented as the mean ± SD. In panel (**a**), ** *p* < 0.01, *** *p* < 0.001 compared with the LPS group. In panel (**b**), * *p* < 0.05, ** *p* < 0.01, *** *p* < 0.001 compared with the EP group. UA, Urolithin A; RAW264.7, mouse macrophages; IL-6, interleukin-6; TNF-α, tumor necrosis factor-a; LPS, lipopolysaccharide; SD, standard deviation; NG, Negative Control group; EP, Experimental Periodontitis group; DX, Doxycycline group; UL, Low-dose UA group; UH, High-dose UA group.

Based on the results of the Enzyme-Linked Immunosorbent Assay (ELISA) results, the UA group exhibited a significant dose-dependent anti-inflammatory effect compared with the lipopolysaccharide (LPS) group (Figure 2a). The levels of inflammatory cytokines were significantly reduced in the 30 μM UA group, indicating that 30 µM UA effectively inhibited the LPS-induced inflammatory response. Therefore, UA could significantly downregulate the secretion of interleukin-6 (IL-6) and tumor necrosis factor-alpha (TNF-α), two major pro-inflammatory cytokines, in a dose-dependent manner, providing direct experimental evidence for its anti-inflammatory effects. Meanwhile, the changes in IL-6 and TNF-α in in vivo experiments were consistent with those in the in vitro investigation. Significantly elevated serum levels of TNF-α and IL-6 were detected in the Experimental Periodontitis (EP) group, with significant differences compared to the Negative Control (NG) group (Figure 2b). In the UA treatment groups, the High-dose UA (UH) group exhibited the best anti-inflammatory effect, showing significant differences compared to the EP group. Notably, there was no significant difference in the comparison of these levels between the UH group and the NG group, indicating that the high dose of UA can effectively inhibit the systemic inflammatory response. These results further confirmed the anti-inflammatory effects of UA in vivo and supported its potential application as a therapeutic agent for periodontitis.

**Figure 3:**
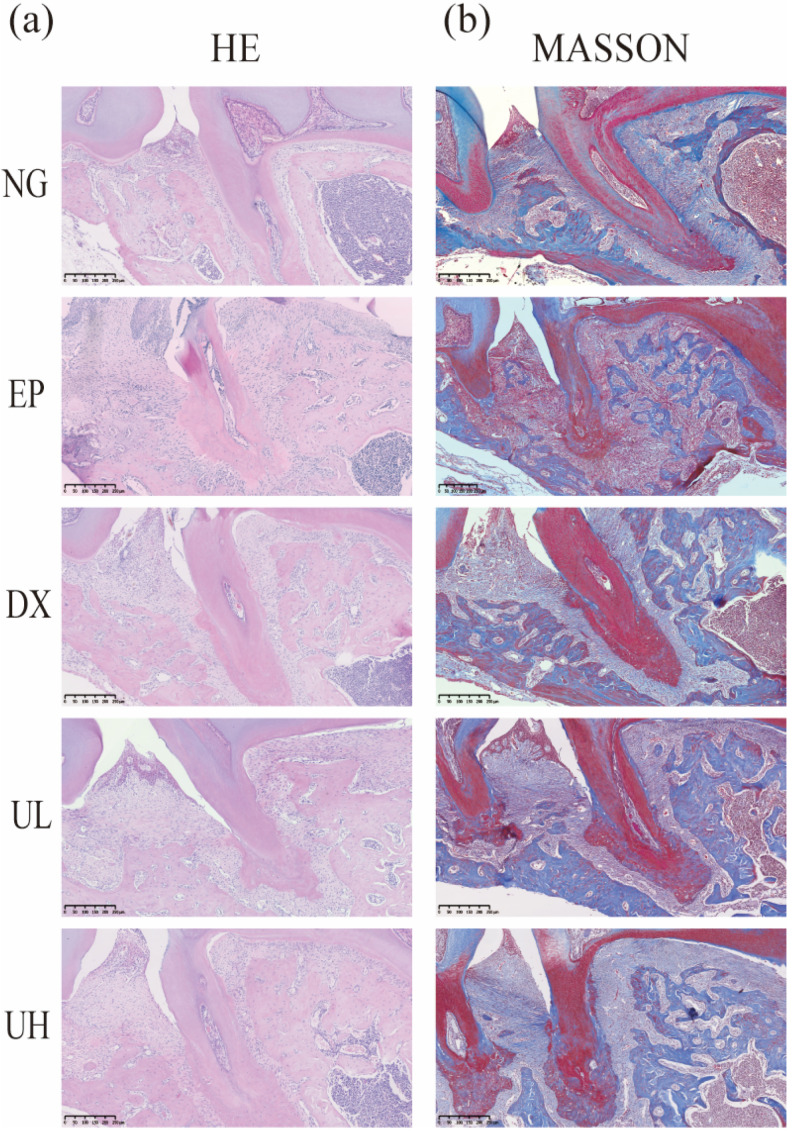
Pathological alterations in periodontal tissues. (**a**) H&E staining of coronal sections from decalcified, paraffin-embedded maxillary tissue samples. (**b**) Periodontal tissues stained with Masson. H&E, Hematoxylin, and eosin; Masson, Masson’s trichrome; NG, Negative Control group; EP, Experimental Periodontitis group; DX, Doxycycline group; UL, Low-dose UA group; UH, High-dose UA group.

According to hematoxylin and eosin (H&E staining) (Figure 3a), the NG group exhibited intact gingival epithelial structure and normal alveolar bone height. In contrast, the EP group showed obvious alveolar bone resorption, confirming successful periodontitis modeling. Compared to the EP group, the Doxycycline (DX) group, and Low-dose UA (UL) group showed a less severe degree of alveolar bone resorption. Notably, the UH group revealed the best therapeutic effect, exhibiting the lowest degree of alveolar bone resorption. Accordingly, high-dose UA could alleviate bone resorption. Furthermore, Masson’s trichrome (Masson) trichrome staining results validated the reparative effects of UA on periodontal tissue (Figure 3b). To be specific, the NG group showed intact periodontal tissue structure, regularly and orderly arranged collagen fibers, and extension of fiber bundles from the cementum to the alveolar bone, forming a typical “fan-shaped” structure. The EP group showed disorganized collagen fiber arrangement, fiber bundle rupture, and widening of the periodontal membrane space. In comparison with the EP group, the DX group and UL group showed more a regular arrangement of collagen fibers. The UH group exhibited minimal fiber disorganization and degeneration, with newly formed collagen fibers extending from the cementum of the first molar to the cementum of the second molar, indicating enhanced periodontal tissue repair. Collectively, UA, especially high-dose UA, can effectively promote collagen fiber reconstruction and repair in periodontal tissue.

### 2.3. Effect of UA on Alveolar Bone Resorption in a Periodontitis Model

**Figure 4:**
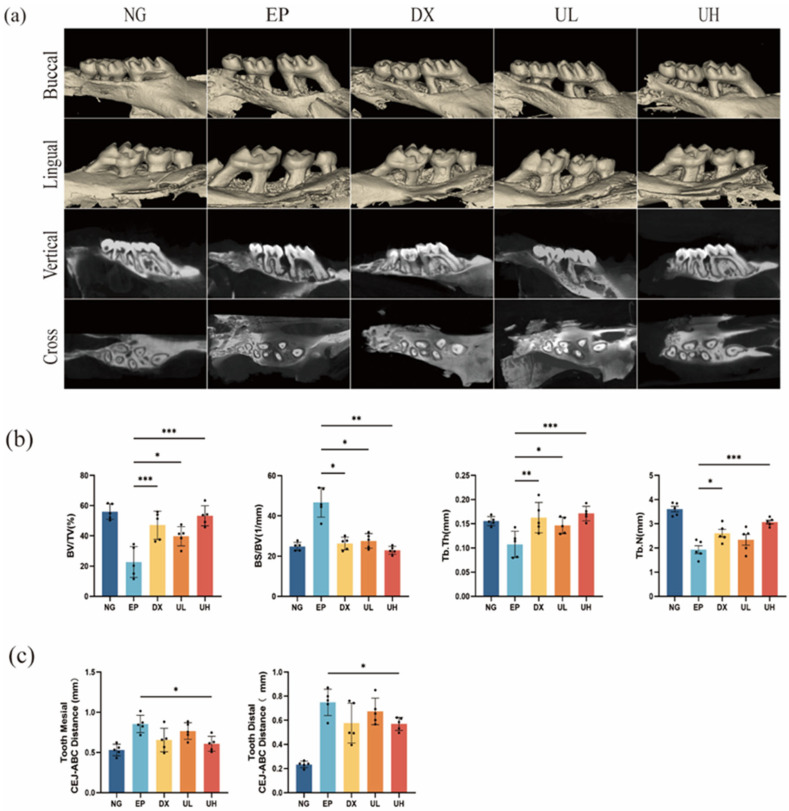
Effect of UA on teeth and periodontal tissues in a mouse periodontitis model assessed by Micro-CT. (**a**) Micro-CT analysis showing the maxillae’s three-dimensional reconstructed images following various treatments. (**b**) Quantitative analysis of BV/TV, BS/BV, Tb.Th, and Tb.N. (**c**) Measurement of mesial and distal CEJ–ABC distances. Data are presented as the mean ± SD. * *p* < 0.05, ** *p* < 0.01, *** *p* < 0.001 compared with the EP group. UA, Urolithin A; Micro-CT, Micro-computed tomography; BV/TV, bone volume/total tissue volume; BS/BV, bone surface area/bone volume; Tb.Th, trabecular thickness; Tb.N, trabecular number; CEJ–ABC, alveolar bone crest–cementoenamel junction; SD, standard deviation; NG, Negative Control group; EP, Experimental Periodontitis group; DX, Doxycycline group; UL, Low dose UA group; UH, High dose UA group.

UA significantly inhibited ligature-induced alveolar bone resorption, as evidenced by Micro-computed tomography (Micro-CT) analysis (Figure 4a). Compared to the NG group, the EP group exhibited significant alveolar bone resorption at both the mesial and distal aspects of the first molar, with a significant increase in the cemento-enamel junction to the alveolar bone crest (CEJ-ABC) distance (Figure 4c). Meanwhile, compared to the EP group, significantly reduced CEJ-ABC distance was observed in the DX group and UA treatment groups (UL and UH), particularly in the UH group. Further morphometric analysis indicated that compared to the EP group, UA treatment groups had obviously improved bone microstructural parameters (e.g., bone volume fraction (BV/TV), bone surface-to-volume ratio (BS/BV), trabecular thickness (Tb.Th), and trabecular number (Tb.N), revealing better bone protective effect of high-dose UA (Figure 4b). Altogether, UA can effectively inhibit periodontitis-related alveolar bone resorption and improve bone microstructure in a dose-dependent manner.

**Figure 5:**
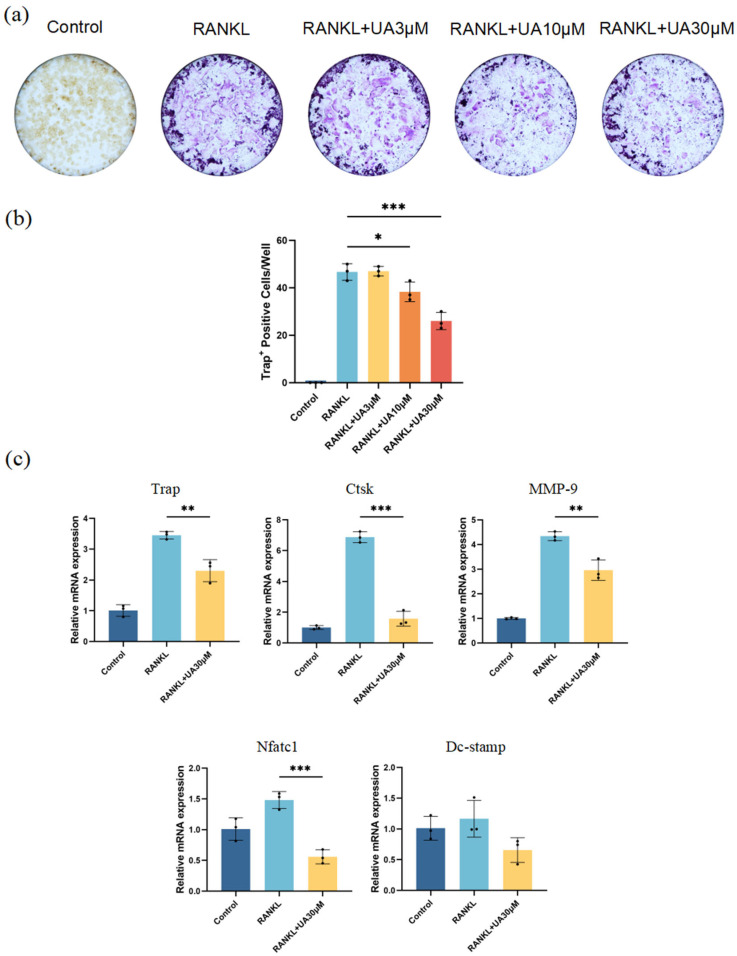
UA inhibits osteoclast differentiation. (**a**) TRAP staining images; (**b**) Quantification of osteoclast numbers. (**c**) Effect of UA on the relative mRNA expression levels of TRAP, Ctsk, MMP-9, Nfatc1, and DC-Stamp after RANKL-induced osteoclast differentiation of RAW264.7 cells. Data are presented as the mean ± SD. * *p* < 0.05, ** *p* < 0.01, *** *p* < 0.001 compared with the RANKL group. UA, Urolithin A; TRAP, tartrate-resistant acid phosphatase stain; Ctsk, cathepsin K; MMP-9, matrix metalloproteinase-9; Nfatc1, nuclear factor of activated T cells 1; DC-Stamp, dendritic cell-specific transmembrane protein.

In vitro cell experiments revealed that compared with the control group, treatment with UA at various concentrations resulted in a remarkably reduced number and area of osteoclasts (Figure 5a). The most significant inhibitory effect was observed for UA at 30 μM (Figure 5b). Therefore, UA might suppress nuclear factor-κB ligand (RANKL)-induced osteoclast differentiation of mouse macrophages (RAW 264.7) in a dose-dependent manner by reducing tartrate-resistant acid phosphatase (TRAP) activity. Furthermore, quantitative real-time polymerase chain reaction (qRT-PCR) analysis demonstrated that treatment with 30 μM UA significantly downregulated the mRNA expression of TRAP, cathepsin K (Ctsk), nuclear factor of activated T cells 1 (NFATc1), and matrix metalloproteinase-9 (MMP-9). Notably, UA treatment also reduced the expression level of dendritic cell-specific transmembrane protein (DC-Stamp), yet without statistically significant difference (Figure 5c).

**Figure 6:**
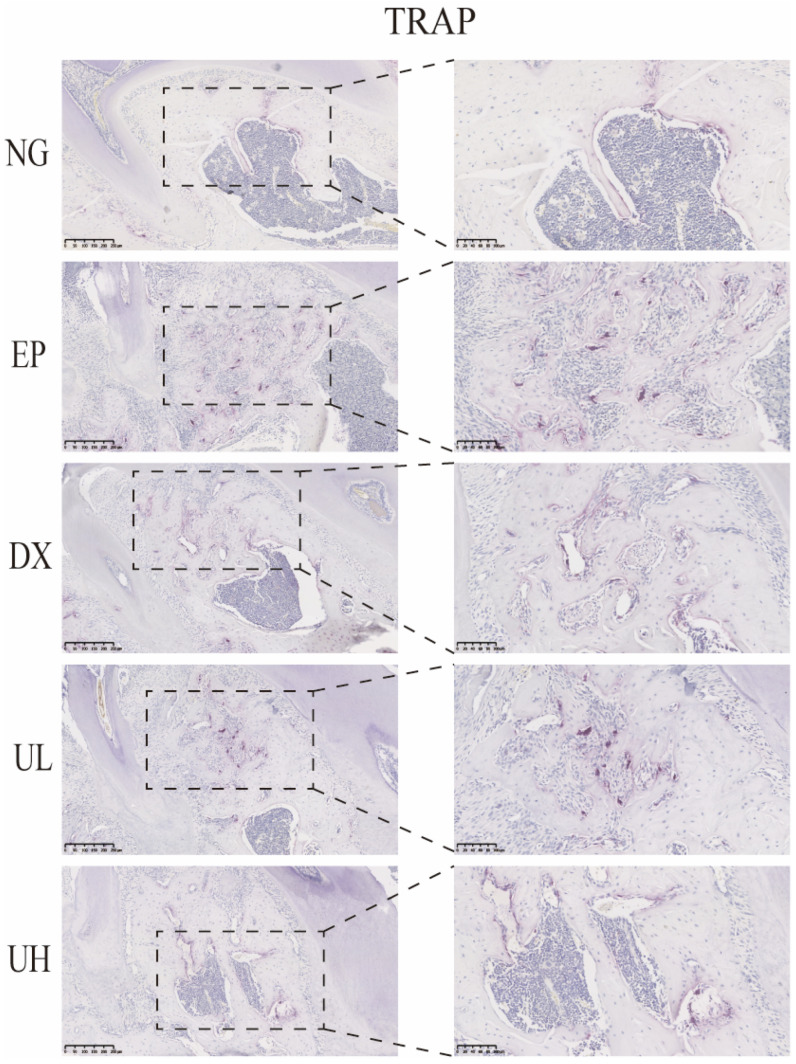
UA inhibits osteoclast activity in periodontitis. TRAP staining showed a reduction in osteoclast formation in the DX, UL, and UH groups. UA, Urolithin A; TRPA, tartrate-resistant acid phosphatase; NG, Negative Control group; EP, Experimental Periodontitis group; DX, Doxycycline group; UL, Low dose UA group; UH, High dose UA group.

Similarly, in vivo TRAP staining results also demonstrated that UA significantly inhibited the activation of periodontitis-induced osteoclasts (Figure 6). The EP group exhibited a large number of TRAP-positive multinucleated osteoclasts in the first molar region, primarily distributed at the sites of alveolar bone resorption lacunae, significantly increased when compared to the NG group. When compared to the EP group, the DX group and UA treatment groups (UL and UH) showed a significant reduction, especially in the UH group. This observation was in good agreement with the degree of bone resorption shown by Micro-CT, further confirming that UA reduces alveolar bone resorption by inhibiting osteoclast activity.

**Figure 7:**
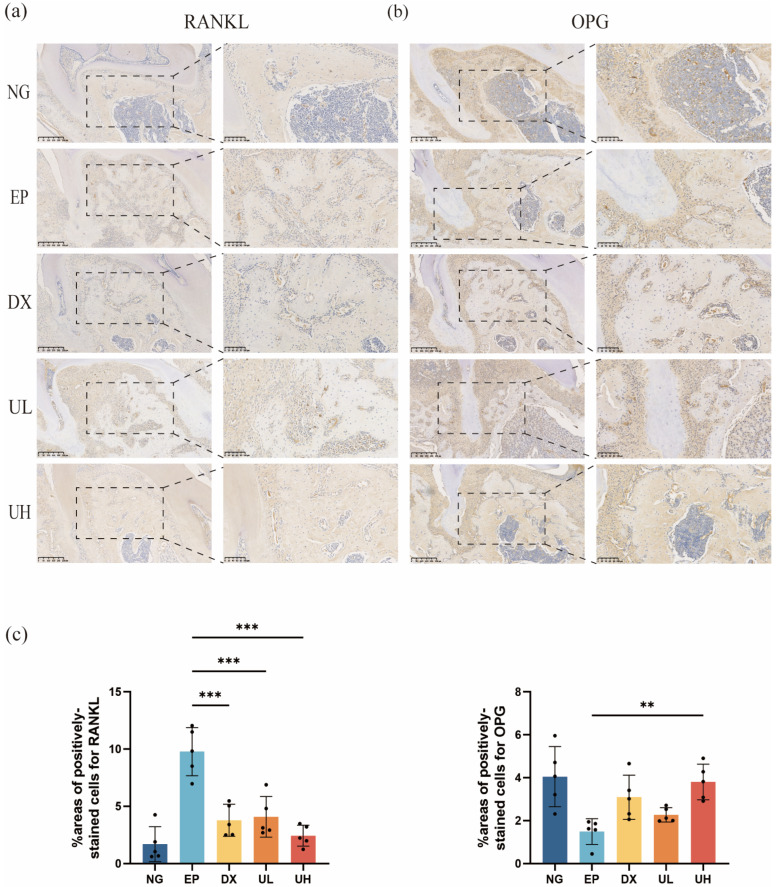
UA Inhibits Periodontitis Progression. (**a**) Immunohistochemical staining of periodontal tissues for RANKL expression. (**b**) Immunohistochemical staining of periodontal tissues for OPG expression. (**c**) The % area of cells stained positive for RANKL and OPG were counted. Data are presented as the mean ± SD. ** *p* < 0.01, *** *p* < 0.001 compared with the EP group. UA, Urolithin A; RANKL, Nuclear factor-kB ligand; OPG, Osteoprotegerin; SD, standard deviation; NG, Negative Control group; EP, Experimental Periodontitis group; DX, Doxycycline group; UL, Low dose UA group; UH, High dose UA group.

In addition, immunohistochemical analysis suggested a remarkable regulatory role of UA in the balance of the RANKL/OPG system. Compared to the NG group, the EP group exhibited a significantly upregulated RANKL expression (Figure 7a) and downregulated OPG expression (Figure 7b) in periodontal tissues. The DX and UL groups showed a significant reduction in RANKL expression but no significant difference in OPG expression compared to the EP group. In contrast, in the UH group, RANKL expression was significantly reduced compared to the EP group, and OPG expression was significantly increased, without significant difference compared to the NG group (Figure 7c). To this end, our investigation deciphered the mechanism of UA in suppressing osteoclast activation and alveolar bone resorption at the molecular level. High-dose UA could effectively restore RANKL/OPG system balance, providing new evidence for its anti-inflammatory and bone-protective properties.

## 3. Discussion

UA is a natural polyphenol metabolite derived from ETs, which are abundant in pomegranates, strawberries, raspberries, walnuts, etc. UA is produced by the gut microbiota through the microbial metabolism of dietary ETs, exhibiting diverse pharmacological properties. UA can exert its anti-inflammatory effects by activating the AMP-activated protein kinase (AMPK) signaling, a pathway with prominent roles in regulating the inflammatory response of various immune cells, including T cells, mast cells, neutrophils, and macrophages [33,34]. UA was reported to reduce triglyceride accumulation in adipocytes and hepatocytes through an AMPK-dependent pathway [35]. Its anti-inflammatory mechanisms mainly include: (1) inactivation of NF-κB and its nuclear translocation to inhibit the levels of pro-inflammatory cytokines such as IL-1β, IL-6, and TNF-α [36]. (2) inhibition of MAPK signaling pathway phosphorylation, regulating the pro-inflammatory network and cytokine biosynthesis [22]. All these molecular data support the application of UA in treating inflammation-related diseases. Concerning osteoclast inhibition, Liu et al. demonstrated that UA could stimulate Wnt expression, activating GSK3β, thereby regulating osteogenic marker transcription [37]. Fu et al. documented the potential of UA as a therapeutic agent for osteoarthritis, considering that it could effectively inhibit IL-1β-induced activation of the PI3K/Akt/NF-κB pathway, thereby reducing inflammation and catabolic processes in osteoarthritic chondrocytes [38]. In another study carried out by Wei et al., UA was observed to suppress osteoclastogenesis through dual mechanisms: (1) blocking NF-κB and p38 MAPK signaling pathways and (2) activating the Nrf2/HO-1 antioxidant stress axis, thereby inhibiting RANKL-induced osteoclast differentiation [39]. Despite the confirmed important role of UA in bone metabolism regulation, there is so far limited research on its application in the treatment of periodontitis. For the first time, this study systematically explored the therapeutic effects of UA on experimental periodontitis and its potential mechanisms, with the purpose of offering additional experimental evidence for developing UA-based therapeutic strategies for periodontitis.

In this study, we successfully established an experimental periodontitis model in C57BL/6 mice. According to the histological analysis, the EP group showed significant pathological features, providing an ideal experimental platform for evaluating the therapeutic effects of UA. In general, the pathological process of periodontitis involves complex host-microbe interactions. Toxins, antigenic components, and metabolic products produced by periodontal pathogens can stimulate epithelial cells to release various inflammatory factors (e.g., IL-1β, IL-6, and TNF-α) [40]. IL-6 participates in the pathogenesis of periodontitis through immune regulation as an important pro-inflammatory or by upregulating RANKL expression in osteoblasts to promote osteoclast differentiation and bone resorption [41]. IL-6 has been recognized as a key pro-inflammatory and bone resorption-related cytokine as it is widely expressed in various cell types and can directly regulate immune responses and osteoclast activity. A large number of in vitro and in vivo studies have confirmed that IL-6 was significantly upregulated under inflammatory stimulation and obviously downregulated after effective treatment [42]. Therefore, IL-6 may be involved critically in the pathological process of periodontitis. Similarly, TNF-α is also a major pro-inflammatory factor that drives inflammatory response and stimulates the release of other inflammatory mediators, thereby exacerbating the destruction of periodontal tissues. Meanwhile, TNF-α influences bone metabolism by promoting RANK expression in osteoclast precursors or upregulating RANKL expression in osteoblasts. Despite no direct impact on osteoclast differentiation, they can significantly enhance the RANKL/RANK signaling pathway, thereby promoting osteoclast activation and bone resorption [43]. This study confirmed the anti-inflammatory effect of UA in vitro and in vivo. In the in vitro experiments, LPS stimulation significantly promoted TNF-α and IL-6 secretion in RAW264.7 cells, while UA pretreatment at 10 and 30 µM dose-dependently inhibited this effect, resulting in decreased TNF-α and IL-6 levels. In the in vivo experiments, serum levels of TNF-α and IL-6 were significantly higher in the EP group compared to the NG group but obviously reduced in the DX group and UA-treated groups (UL and UH), particularly in the UH group. Therefore, UA can inhibit the release of inflammatory mediators in a concentration-dependent manner, alleviating the inflammatory response in periodontal tissues and slowing the tissue destruction process.

In our study, an in vitro model for osteoclastogenesis was established using the RAW264.7 cells to investigate the inhibitory effect of UA on osteoclast differentiation. RAW264.7 cells, under RANKL stimulation, can be effectively induced to differentiate into mature osteoclasts with bone-resorbing activity. This model has been widely used in studies of bone metabolic disorders such as osteoporosis, rheumatoid arthritis, pathological bone resorption, and periodontitis [44,45]. TRAP staining is a classical method for assessing osteoclast differentiation, enabling the evaluation of bone-resorbing activity by visualizing and quantifying TRAP-positive cells. Therefore, initially, in our experiment, TRAP staining was applied for osteoclast phenotype assay. Upon treatment with UA at different concentrations, it was observed with markedly reduced number and size of osteoclasts, as well as TRAP activity, with the most pronounced inhibitory effect observed at 30 μM UA.

Subsequently, the optimal UA concentration identified from TRAP staining was determined for qRT-PCR to further explore the effect of UA on the expression of osteoclast-related genes. UA significantly downregulated the mRNA expression of TRAP, NFATc1, and MMP-9, yet without significant effect on DC-Stamp expression. A series of genes (i.e., TRAP, NFATc1, DC-Stamp, Ctsk, and MMP-9) involved in osteoclast formation and function are upregulated during the RANKL-induced differentiation of RAW264.7 cells into osteoclasts. All these genes play key roles in bone resorption and degradation, thus contributing to bone remodeling and skeletal homeostasis.

Two key transcription factors required for osteoclastogenesis are Cellular FBJ Osteosarcoma Oncogene (c-Fos) and NFATc1. In osteoclast precursors, NFATc1 may be activated by the calcium signaling, which cooperates with the AP-1 complex to amplify its own expression through a positive feedback loop [46], subsequently initiating the transcription of osteoclast-specific genes such as TRAP, Ctsk, and MMP-9 during terminal differentiation [47]. Specifically, TRAP is a classical osteoclast marker possessing lysosomal activity directly involved in bone resorption [47]; Ctsk, a cysteine protease specifically expressed in osteoclasts, is essential for bone matrix degradation, and its deficiency may result in impaired bone resorption and osteopetrosis [48,49]. MMPs belong to the zinc-dependent endopeptidase family and have the ability to degrade extracellular matrix components in various tissues, including bone. MMP-9, with high expression in osteoclasts, participates significantly in extracellular matrix degradation and bone remodeling during increased osteoclast activity [50]. DC-Stamp, on the other hand, can regulate the fusion of osteoclast precursors and is essential for the formation of multinucleated osteoclasts. Osteoclasts isolated from DC-Stamp-knockout mice were observed to exhibit impaired cell–cell fusion, resulting in mononuclear cells and a mild osteopetrotic phenotype [51,52].

With respect to the above, UA could downregulate the expression of TRAP and other osteoclast-related genes during osteoclast differentiation induced by RANKL in RAW264.7 cells, thereby inhibiting osteoclastogenesis and potentially promoting bone remodeling, yet with the precise mechanisms remain to be elucidated. These results were further supported by TRAP staining of osteoclasts in vivo. The RANKL/RANK/OPG signaling pathway is a key regulator of osteoclast activation and differentiation [53]. RANKL is a key factor in osteoclast differentiation, which has been confirmed at the genetic level that RANKL gene knockout mice or individuals carrying RANKL gene mutations fail to form functional osteoclasts, exhibiting phenotypes of osteosclerosis and abnormally increased bone mass [54]. It highlights the indispensability of RANKL in osteoclastogenesis and also offers important clues for understanding the regulatory mechanisms of bone metabolism. Furthermore, OPG is secreted by osteoblasts and stromal cells, featured distinctively by the absence of a transmembrane domain but possessing a domain similar to RANK, enabling it to function as a “decoy receptor” for RANKL. OPG can competitively suppress the binding of RANKL to RANK, effectively blocking osteoclast activation and inhibiting the bone resorption process. Therefore, OPG is considered a protective factor against osteoporosis [55]. During periodontal disease, the RANKL/OPG ratio has been recognized as an important biomarker for evaluating the progression of periodontitis, considering that an elevated ratio is positively correlated with the degree of alveolar bone resorption [56]. Therefore, regulating the RANKL/RANK/OPG signaling pathway may be a feasible strategy for treating periodontitis-related bone resorption. In this study, UA could modulate the RANKL/RANK/OPG signaling pathway and inhibit osteoclast-mediated bone resorption. Both in vivo and in vitro experiments showed a significantly reduced number of osteoclasts. Additionally, RANKL expression was significantly decreased in the DX, UL, and UH groups compared to the EP group, while OPG expression showed a dose-dependent increase. Consequently, UA can inhibit alveolar bone resorption by downregulating RANKL expression and upregulating OPG expression, thus restoring the RANKL/OPG balance and reducing osteoclast formation.

Through systematic analyses, this study compared the therapeutic effects of UA and doxycycline on experimental periodontitis. At the same dosage, UA exhibited slightly less efficacy than doxycycline in alleviating periodontitis-induced inflammation, inhibiting bone resorption, and promoting tissue repair. The most pronounced therapeutic effects of UA were observed at higher concentrations, displaying a clear dose-dependent response that was consistently confirmed in both in vivo and in vitro models. Furthermore, histological and Micro-CT analyses of alveolar bone morphology using H&E and Masson staining further confirmed the dose-dependent protective effect of UA on alveolar bone. Considering the well-established efficacy of doxycycline in non-surgical periodontal therapy and corresponding results in this study, it can be inferred that UA exerts its therapeutic effects through the following mechanisms: (1) significantly inhibiting the production of pro-inflammatory factors (e.g., IL-6 and TNF-α); (2) suppressing osteoclastogenesis; and (3) regulating the balance of RANKL/OPG expression. On this basis, UA may be useful for preventing and treating osteoporosis and periodontitis-related bone loss, providing important experimental evidence for developing novel therapeutic strategies targeting UA.

Notably, this study still has several limitations that merit attention. First, this study only adopted mouse cell lines, necessitating the use of more cell lines and human primary cultured cells to further prove the protective effects of UA. Second, our experiments only evaluated the cytotoxicity of UA in vitro, with a lack of assessing its long-term safety and toxicity in vivo. Critically, the long-term toxicity and pharmacological efficacy of UA require further validation through in vivo experiments despite its favorable safety profile confirmed in in vitro and in existing animal studies [15]. The safety of UA should be thoroughly evaluated through the incorporation of comprehensive preclinical models in future studies. Next, the ligature-induced periodontitis model in mice, even with wide application, does not fully replicate the complex and chronic microbial–immune interactions in human periodontitis. Finally, although our study supports UA as a promising therapeutic agent for periodontitis, caution should be exercised when interpreting our findings based on a short-term (2-week) intervention, as periodontitis is a chronic disease. Further long-term studies are warranted to evaluate the sustained efficacy and safety of UA for the treatment of chronic periodontitis.

## 4. Materials and Methods

### 4.1. Reagents and Antibodies

Dulbecco’s Modified Eagle Medium (DMEM), alpha Minimum Essential Medium (α-MEM), fetal bovine serum (FBS), and penicillin-streptomycin solution were purchased from Thermo Fisher Scientific (Waltham, MA, USA). UA [Cat# CYK-Z2526, with purity > 95–99% determined by high-performance liquid chromatography (HPLC)] was obtained from Chengdu Grass Source Kang Biotechnology Co., Ltd. (Chengdu, China). Doxycycline (Cat# HY-N0565, purity > 98% by HPLC) and sodium carboxymethyl cellulose (CMC-Na) were supplied by MedChemExpress (Monmouth Junction, NJ, USA). Atropine sulfate was purchased from Chengdu Brilliant Pharmaceutical Co., Ltd. (Chengdu, China). The CCK-8 assay kit and Calcein-AM/Propidium Iodide (PI) double staining kit were purchased from Beyotime Biotechnology Co., Ltd. (Shanghai, China). Lipopolysaccharide (LPS), hematoxylin and eosin (H&E), Masson’s trichrome, tartrate-resistant acid phosphatase (TRAP) staining kit, and 5% goat serum were obtained from Beijing Solarbio Science & Technology (Beijing, China). Zoletil 50 was obtained from Virbac S.A. (Carros, France). RNAiso Plus (Cat# 9018), PrimeScript™ RT reagent kit with gDNA Eraser (Perfect Real Time) (Cat# RR047A), and TB Green^®^ Premix Ex Taq™ II (Tli RNaseH Plus) (Cat# RR820A) were all purchased from Takara Bio Inc. (Shiga, Japan). PCR primers were synthesized by Sangon Biotech Co., Ltd. (Shanghai, China). Enzyme-linked immunosorbent assay (ELISA) kits were provided by MultiSciences (Lianke) Biotech Co., Ltd. (Hangzhou, China). The primary antibody against receptor activator of nuclear factor-κB ligand (RANKL) (Cat# 23408-1-AP) and secondary antibodies (Cat# RGAU011) were purchased from Proteintech Group, Inc. (Wuhan, China). The antibody against Osteoprotegerin (OPG) (Cat# bs-20624R) was obtained from Biosynthesis Biotechnology (Beijing, China). The 3,3’-Diaminobenzidine (DAB) substrate kit (Cat# ZLI-9018) was obtained from ZSGB-BIO (Beijing, China). RANKL osteoclast induction medium (Cat# 462TEC010) was obtained from R&D Systems (Minneapolis, MN, USA).

### 4.2. Cell Culture

After thawing, mouse fibroblasts (L929) and RAW 264.7 macrophages (Pricella, Wuhan, China) were resuspended in a complete culture medium consisting of DMEM supplemented with 10% FBS and 1% penicillin-streptomycin solution. After mixing thoroughly, the cell suspension was centrifuged at 1200 rpm for 3 min, followed by resuspension of the cell pellet in a fresh, complete medium when the supernatant was discarded. Cells were cultured in a humidified incubator at 37 °C with 5% CO_2_, with the medium replaced every two days. Cells were passaged upon reaching 80% confluence, and cells from passages 2 to 3 were used for subsequent experiments.

### 4.3. Cell Viability Assay

The effect of UA on cell viability was assessed using the CCK-8 assay. Cells were first separated using trypsin and then resuspended in fresh media to seed L929 cells into 96-well plates at a density of 1 × 10^4^ cells per well. Following a 24-h incubation period, cells with changed culture medium were divided into the blank (medium only), control (untreated cells), and UA-treated (UA at 3, 10, 30, 50, 100, and 500 μM for 24, 48, and 72 h) groups, with five replicate wells per group. After that, a 10% CCK-8 solution was prepared to assess cell viability according to the manufacturer’s instructions. After cell rinsing with phosphate-buffered saline (PBS), 100 µL of the freshly prepared CCK-8 solution was added to each well for 2 h of cell incubation at 37 °C. A microplate reader was used to measure absorbance. Finally, the absorbance at 450 nm was measured using a microplate reader (Synergy H1, BioTek Instruments, Winooski, VT, USA).

### 4.4. Calcein-AM/Propidium Iodide(PI) Staining

L929 cells were plated into 24-well plates (1 × 10^4^ cells/well) and incubated for 24 h to allow for cell adhesion. Subsequently, UA was added at final concentrations of 3, 10, 30, 50, and 100 μM in the complete medium. A control group was established with the addition of an equivalent volume of fresh culture medium. With the aspiration of the medium after another 24 h of incubation, each well was rinsed twice with PBS to remove non-adherent cells and residual compounds. Then, cell viability was assessed using a Calcein-AM/PI double staining kit. A working solution was prepared by mixing Calcein-AM, PI, and PBS in a 1:1:1000 volume ratio. Following the supplementation of 250 μL of this staining solution to each well, incubation was performed at 37 °C in the dark for 20 min. Living cells (Calcein-AM positive) were stained green, while dead cells (PI positive) appeared red. Images were captured using an inverted fluorescence microscope (Axio Observer 3, Carl Zeiss AG, Oberkochen, Germany).

### 4.5. Measurement of TNF-α and IL-6 Release by Enzyme-Linked Immunosorbent Assay (ELISA)

For in vitro investigation, RAW 264.7 cells were seeded in 24-well plates at 5 × 10^4^ cells per well. In order to evaluate the anti-inflammatory effects of UA, three groups were established, including control, LPS, and UA groups. The UA group was pretreated with UA (3, 10, or 30 μM) for 2 h, and then both the LPS and UA groups were stimulated with 1 μg/mL LPS for 24 h; while the control group received only medium changes without any treatment. After centrifugation, the supernatant was collected for further analysis. In terms of in vivo experiments, mice were anesthetized with atropine sulfate and Zoletil 50. To separate the serum, 1.5 mL of blood samples were drawn from the abdominal aorta of mice and centrifuged for 10 min at 2000 rpm. ELISA kits were used to measure the serum levels of TNF-α and IL-6. The absorbance at 450 nm was measured using a microplate reader to compute the cytokine concentrations. Finally, the cytokine concentrations were computed through the measurement of the absorbance at 450 nm using a microplate reader (Synergy H1, BioTek Instruments, Winooski, VT, USA).

### 4.6. Osteoclast Differentiation

RAW264.7 cells were seeded into 96-well plates (3 × 10^3^ cells/well) to evaluate the inhibitory effect of UA on osteoclast differentiation. After 24 h of incubation to allow full adherence, the DMEM complete medium was replaced with α-MEM complete medium. Then, the cultured cells were randomly divided into the following three groups: (1) Control group: replacement of the medium with fresh α-MEM complete medium only; (2) RANKL group: supplementation with 50 ng/mL RANKL to induce osteoclast differentiation; and (3) RANKL + UA group: treatment with 100 ng/mL RANKL and varying concentrations of UA (3, 10, and 30 μM). Three replicate wells were set for each group. On day 5 of culture, TRAP staining was performed to assess osteoclast differentiation. TRAP staining solution was prepared according to the manufacturer’s instructions. Cells were first washed thoroughly with PBS, then fixed with 4% paraformaldehyde at room temperature for 10 min, followed by another PBS washing. Subsequently, 100 μL of TRAP staining working solution was added to each well for another incubation at 37 °C in the dark for 30 min. After that, the wells were rinsed three times with deionized water and air-dried. Finally, osteoclast differentiation was evaluated and imaged under an inverted fluorescence microscope (Axio Observer 3, Carl Zeiss AG, Oberkochen, Germany).

### 4.7. Quantitative Real-Time Polymerase Chain Reaction (qRT-PCR)

Osteoclasts cultured as described in Section 4.6 were used to assess the mRNA expression levels of osteoclast-specific genes by PCR, including TRAP, nuclear factor of activated T cells 1 (NFATc1), dendritic cell-specific transmembrane protein (DC-Stamp), cathepsin K (Ctsk), and matrix metalloproteinase-9 (MMP-9). Total RNA was extracted from the osteoclast differentiation model using RNAiso Plus (1 mL lysis buffer with 200 μL chloroform). The RNA concentration and purity were determined using a NanoDrop spectrophotometer (ND-2000C, Thermo Fisher Scientific, Waltham, MA, USA). Reverse transcription was performed using the PrimeScript™ RT reagent kit with gDNA Eraser (Perfect Real Time) in accordance with the manufacturer’s specification. qRT-PCR was conducted using the TB Green^®^ Premix Ex Taq™ II kit on a LightCycler^®^ 480 II system (Roche, Basel, Switzerland). Relative mRNA expression levels were calculated using the 2^−ΔΔCt^ method and normalized to β-actin. The primer sequences used for osteoclast marker genes are shown in Table 1.

### 4.8. Animals

Animal experiments were carried out in 30 6-week-old male C57BL/6 mice (21 ± 2 g, pathogen-free, No. SYXK(Zhe)2021-0012) housed in plastic cages under controlled temperature, with ad libitum access to water and normal laboratory chow. Five mice were assigned to the Negative Control group (NG), and the remaining 25 mice underwent experimental periodontitis induction for 8 weeks according to the following protocol. On day 1, mice were anesthetized via intraperitoneal injection of atropine sulfate and Zoletil 50. Then, mice were tied around the cervical portion of the first molar in the left maxilla using a 6-0 silk thread, which was secured by tying a knot on the vestibular side. During the entire period of experimental periodontitis induction, mice were provided with a sucrose solution (100 g/L) via drinking water. After 8 weeks, twenty mice were then randomly selected and divided into four groups (n = 5/group): Experimental Periodontitis (EP), Doxycycline (DX), Low-dose UA (UL), and High-dose UA (UH) groups. Mice in the DX group received doxycycline (10 mg/kg/day) dissolved in 0.5% CMC-Na via daily intragastric administration for two weeks. This dosage was selected based on previous studies demonstrating its anti-inflammatory and bone-protective effects in experimental periodontitis models without inducing significant systemic toxicity [57,58]. Similarly, mice in the UL and UH groups were given UA (10 mg/kg/day and 20 mg/kg/day, respectively) in 0.5% CMC-Na via daily intragastric administration for two weeks. The doxycycline dose was aligned with that of the low-dose UA group (10 mg/kg) to enable a direct comparison of the therapeutic efficacy between the two compounds. In accordance with the ARRIVE (Animal Research: Reporting of Experiments in Vivo) guidelines, all experimental protocols related to animals were approved by the Animal Ethical and Welfare Committee of Zhejiang Chinese Medical University (No. IACUC-202403-11).

### 4.9. Micro-Computed Tomography

Mice were humanely euthanized using cervical dislocation and subsequently scanned under Micro-CT (Bruker, Billerica, MA, USA) to observe the left maxillary alveolar bone of each group. The acquired images were reconstructed using CTvox 3.3.1 (Bruker) software. Next, our study analyzed the following parameters of the distance from the cemento-enamel junction to the alveolar bone crest of the upper first molar, BV/TV, BS/BV, Tb.Th, and Tb.N using CTAn (Bruker). In addition, the 3D model was reconstructed using Mimics 21.0 (Materialise, Leuven, Belgium) software.

### 4.10. Haematoxylin and Eosin, Masson and Tartrate-Resistant Acid Phosphatase Staining

After fixation for 48 h in 10% formalin, the maxillary bone tissue block was then decalcified for 3 weeks at room temperature in 0.5 M EDTA, with the decalcifying solution being replaced three times per week. After decalcification, the tissue was rinsed under running water for 20 min to remove residual decalcification solution. Following trimming into appropriately sized blocks, the specimens were subjected to routine dehydration and embedding in paraffin. Serial sections, approximately 4 μm in thickness, were cut along the proximal-distal-medial axis of the teeth. These sections were stained with H&E, Masson, and TRAP reagents. Finally, a microscopic examination was performed, focusing on the inflammatory cells and pathological changes in the periodontal tissues surrounding the first molar.

### 4.11. Immunohistochemistry

The prepared sections were provided with antigen repair by a 4-h incubation in EDTA Antigen Retrieval Solution (pH 9.0) at 65 °C. Then, to inhibit endogenous peroxidase activity, the next step was incubation for 10 min at room temperature with 3% hydrogen peroxide. Next, sections were incubated with 5% goat serum for 20 min at 37 °C. The primary antibodies against RANKL (1:200) or OPG (1:200) were applied for another overnight incubation at 4 °C. On the subsequent day, the sections were incubated for 1 h with secondary antibodies.

### 4.12. Statistical Analyses

Data in mean ± standard deviation (SD) were analyzed using GraphPad Prism 9.5.0 (GraphPad Software, San Diego, CA, USA). The normality of the data was assessed using the Shapiro–Wilk test, while homogeneity of variance was evaluated by the F-test or the Brown–Forsythe test. Kruskal–Wallis test with Dunn’s correction was used for non-normal data (*p* ≤ 0.05 in Shapiro–Wilk test). Multi-group comparisons employed a one-way analysis of variance, followed by Tukey’s post hoc test when variances were equal or the Games–Howell test when variances were unequal. A *p*-value of <0.05 was considered statistically significant.

## 5. Conclusions

In summary, this study demonstrated that UA could reduce alveolar bone resorption and inflammatory infiltration in an experimental periodontitis mouse model. UA could act as a potential therapeutic agent for the prevention or treatment of periodontitis.

## Figures and Tables

**Table 1:** Sequences of primers used in the gene expression analysis.

Genes	Forward Primer (5′-3′)	Reverse Primer (5′-3′)
Trap	CACTCCCACCCTGAGATTTGT	CATCGTCTGCACGGTTCTG
Ctsk	GAAGAAGACTCACCAGAA-GCAG	TCCAGGTTATGGGCAGAGATT
MMP-9	CTG-GACAGCCAGACACTAAAG	CTCGCGGCAAGTCTTCAGAG
NFATc1	GGAGCGGAGAAACTTTGCG	GTGACACTAGGG-GACACATAACT
DC-Stamp	TACGTGGAGAGAAGCAAGGAA	ACACTGAGACGTGGTTTAGGAAT
β-actin	CATCCGTAAA-GACCTCTATGCCAAC	ATGGAGCCACCGATCCACA

## Data Availability

The datasets generated and/or analyzed during the current study are available from the corresponding author upon reasonable request.

## Abbreviations

The following abbreviations are used in this manuscript:

UA	Urolithin A
CCK-8	Cell counting kit-8
PBS	Phosphate buffered saline
IL-6	Interleukin-6
TNF-α	Tumor necrosis factor-α
IL-1β	Interleukin-1β
L929	Mouse fibroblasts
RAW264.7	Mouse macrophages
LPS	lipopolysaccharid
DMEM	Dulbecco’s modified eagle
ELISA	Enzyme linked immunosorbent assay
qRT-PCR	Quantitative real-time polymerase chain reaction
TRAP	Tartrate-resistant acid phosphatase stain
NFATc1	nuclear factor of activated T cells 1
Ctsk	cathepsin K
MMP-9	matrix metalloproteinase-9
DC-Stamp	dendritic cell-specific transmembrane protein
HE	Hematoxylin and eosin staining
MASSON	Masson’ trichrome stain
RANKL	Receptor activator of nuclear factor-κB ligand
OPG	osteoprotegerin
Micro-CT	Micro computed tomography
BV/TV	Bone volume/total volume
BS/BV	Bone surface area to bone volume ratio
Tb.N	Trabecular number
Tb.Th	Trabecular thickness
CEJ-ABC	Cemento-enamel junction to alveo bone crest
